# Functional analysis of three putative galactofuranosyltransferases with redundant functions in galactofuranosylation in *Aspergillus niger*

**DOI:** 10.1007/s00203-019-01709-w

**Published:** 2019-08-01

**Authors:** Mark Arentshorst, Davina de Lange, Joohae Park, Ellen L. Lagendijk, Ebru Alazi, Cees A. M. J. J. van den Hondel, Arthur F. J. Ram

**Affiliations:** 1 Institute of Biology Leiden, Molecular Microbiology and Biotechnology, Leiden University, Sylviusweg 72, 2333 BE Leiden, The Netherlands; 2 Present Address: Koppert Biological Systems, Veilingweg 14, 2651 BE Berkel en Rodenrijs, The Netherlands; 3 Present Address: Dutch DNA Biotech, Hugo R Kruytgebouw 4-Noord, Padualaan 8, 3584 CH Utrecht, The Netherlands

**Keywords:** Cell wall integrity, Galactofuranose, Galactomannan, Calcofluor white hypersensitive, Glycosylation, Golgi apparatus

## Abstract

Galactofuranose (Gal*f*)-containing glycostructures are important to secure the integrity of the fungal cell wall. Golgi-localized Gal*f*-transferases (Gfs) have been identified in *Aspergillus nidulans* and *Aspergillus fumigatus*. BLASTp searches identified three putative Gal*f*-transferases in *Aspergillus niger*. Phylogenetic analysis showed that they group in three distinct groups. Characterization of the three Gal*f*-transferases in *A. niger* by constructing single, double, and triple mutants revealed that *gfsA* is most important for Gal*f* biosynthesis. The growth phenotypes of the *ΔgfsA* mutant are less severe than that of the *ΔgfsAC* mutant, indicating that GfsA and GfsC have redundant functions. Deletion of *gfsB* did not result in any growth defect and combining *ΔgfsB* with other deletion mutants did not exacerbate the growth phenotype. RT-qPCR experiments showed that induction of the *agsA* gene was higher in the *ΔgfsAC* and *ΔgfsABC* compared to the single mutants, indicating a severe cell wall stress response after multiple *gfs* gene deletions.

**Electronic supplementary material:**

The online version of this article (10.1007/s00203-019-01709-w) contains supplementary material, which is available to authorized users.

## Introduction

Galactofuranose (Gal*f*) is an important constituent of the fungal cell wall (Tefsen et al. 2012; Oka and Goto 2016). Around 5% of the dry weight of the cell wall of *A. fumigatus* consists of Gal*f* (Lamarre et al. 2009) and similar amounts of Gal*f* are expected to be present in other Aspergilli. Gal*f* is the five-membered ring form of galactose and is found in several cell surface fractions. It has been identified as a component of the cell wall galactomannan fraction in *Aspergilli*, as a part of *N-* and *O-*glycans of extracellular proteins, and within glycosphingolipids (Bardalaye and Nordin 1977; Baretto-Bergter et al. 1980; Wallis et al. 1999; Toledo et al. 2007). We previously reported on the identification of several genes involved in the biosynthesis of Gal*f*-containing glycoconjugates in *A. niger*. The genes involved encode a UDP-glucose 4-epimerase (UgeA), a UDP-galactomutase (UgmA), and two UDP-Gal*f*-transporters (UgtA and UgtB) (Damveld et al. 2008; Park et al. 2014, 2015). Several studies in *A. fumigatus* and *A. nidulans* have shown that similar gene sets of UDP-glucose 4-epimerases, UDP-galactomutases, and UDP-Gal*f*-transporters are present in these fungi and are important for Gal*f* biosynthesis (Lee et al. 2014; El-Ganiny et al. 2008, 2010; Schmalhorst et al. 2008; Engel et al. 2009; Afroz et al. 2011). The genes encoding the final step in the synthesis of Gal*f*-glycostructures, the galactofuranosyl (Gal*f*)-transferases, have been identified in *A. nidulans* and *A. fumigatus* (Komachi et al. 2013; Katafuchi et al. 2017). Gal*f*-transferases use UDP-Gal*f* as a nucleotide sugar donor to transfer Gal*f* to glycostructures such as galactomannan, *N*-chains, and *O*-chains. Gal*f*-transferases are predicted to be present in the Golgi as Golgi-localized UDP-Gal*f* transporters with a crucial function in Gal*f*-biosynthesis (Engel et al. 2009; Afroz et al. 2011; Park et al. 2015). Since Golgi-localized transferases are mostly type II transmembrane proteins, Komachi et al. searched for type II transmembrane protein encoding genes in the genome of *A. nidulans* and systematically deleted these genes. Deletion mutants were analyzed for the presence of Gal*f* on glycostructures, resulting in the identification of GfsA (AN8677) being required for galactofuranosylation of *O-*glycans (Komachi et al. 2013). Deletion of the *A. fumigatus* ortholog (GfsA, Afu6g02120) lead to similar reduction in the presence of Gal*f*-antigens in *O*-glycans, indicating that also the *A. fumigatus* ortholog encodes a Gal*f*-transferase (Komachi et al. 2013). GfsA of *A. fumigatus* and *A. nidulans* were shown to be localized in the Golgi via fractionation experiments or via GFP-tagging, respectively (Komachi et al. 2013; Oka 2018). The GfsA protein of *A. fumigatus* was further characterized biochemically and characterized as a β1,5-galactosyltransferase responsible for the biosynthesis of β1,5-galactosylfuranose in the galactofuran side chain of fungal-type galactomannans (Katafuchi et al. 2017).

To examine the involvement of *A. niger* homologs of the *A. nidulans* and *A. fumigatus* Gal*f*-transferases in galactofuranosylation, putative Gal*f*-transferases in *A. niger* were identified by BlastP searches. Three putative Gal*f*-transferases were identified and their possible redundant functions were examined by making single, double, and triple deletion mutants.

## Methods

### Strains and culture conditions

The *Aspergillus niger* strains used in this study are listed in Table 1. Strains were grown on minimal medium (MM) (Bennett and Lasure 1991), containing 1% (w/v) glucose as carbon source or complete medium (CM) containing 0.5% (w/v) yeast extract and 0.1% (w/v) casamino acids in addition to MM. When required, plates were supplemented with 10 mM uridine. 5-fluoroorotic acid selection to obtain *pyrG*^*−*^ strains was performed as described previously (Arentshorst et al. 2012). Calcofluor white (CFW) sensitivity was determined as described (Ram and Klis 2006). The presence of Gal*f* reactive glycoproteins in the culture medium was performed by growing the strains in 25 ml CM in 50 ml tube Greiner tube for 24 h at 30 °C. Cultures were filtered over a Whatman glass microfiber filter and 2 µl medium was spotted on nitrocellulose blotting paper and labeled with the L10 monoclonal anti-Gal*f*-antibody (Heesemann et al. 2011) as described (Park et al. 2014). Fungal transformations were performed according the protoplast method described by Arentshorst et al. (2012).

**Table 1:** Strains used in this study

Strain	Genotype	Description	References
MA169.4	*cspA1, pyrG378, kusA::DR-amdS-DR*	*ku70* disruption in AB4.1	Carvalho et al. (2010)
MA234.1	*cspA1, kusA::DR-amdS-DR*	Restored *pyrG* in MA169.4	Park et al. (2016)
MA87.6	*cspA1, pyrG378, kusA::amdS, ugmA::AOpyrG*	*ΔugmA* in MA70.15	Damveld et al. (2008)
DL1.1	*cspA1, pyrG378, kusA::DR-amdS-DR,* An12g08720*::AOpyrG*	*ΔgfsA* in MA169.4	This study
DL2.8	*cspA1, pyrG378, kusA::DR-amdS-DR,* An04g06900*::AOpyrG*	*ΔgfsC* in MA169.4	This study
DL3.3	*cspA1, pyrG378, kusA::DR-amdS-DR,* An01g09510*::AOpyrG*	*ΔgfsB* in MA169.4	This study
DL4.1	*cspA1, pyrG378, kusA::DR-amdS-DR,* An04g06900*::AOpyrG,* An12g08720*::*hph	*ΔgfsAC*	This study
DL5.1	*cspA1, pyrG378, kusA::DR-amdS-DR,* An12g08720*::AOpyrG,* An01g09510*::*hph	*ΔgfsAB*	This study
DL6.1	*cspA1, pyrG378, kusA::DR-amdS-DR,* An04g06900*::AOpyrG,* An01g09510*::*hph	*ΔgfsBC*	This study
MA314.1	*cspA1, pyrG378, kusA::DR-amdS-DR,* An04g06900*::AOpyrG,* An01g09510*::*hph*, pyrG*^*−*^	*pyrG*^*−*^ mutant derived from DL6.1	This study
MA316.3	*cspA1, pyrG378, kusA::DR-amdS-DR,* An04g06900*::AOpyrG,* An01g09510*::*hph*, pyrG*^*−*^*,* An12g08720*::AOpyrG*	*ΔgfsABC*	This study
MA877.1	*cspA1, pyrG378,* An12g08720*::AOpyrG*	*ΔgfsA, kusA* restored in DL1.1	This study
MA880.1	*cspA1, pyrG378,* An04g06900*::AOpyrG,* An12g08720*::*hph	*ΔgfsAC kusA* restored in DL4.1	This study
MA881.1	*cspA1, pyrG378, An12g08720::AOpyrG,* An01g09510*::*hph	*ΔgfsAB kusA* restored in DL5.1	This study
MA884.1	MA877.1+*gfsA*+pAN8.1	*ΔgfsA*+*gfsA*	This study
MA887.1	MA881.1+*gfsA*+pAN8.1	*ΔgfsAB*+*gfsA*	This study
MA888.4	MA881.1+*gfsB*+pAN8.1	*ΔgfsAB*+*gfsB*	This study
MA885.1	MA880.1+*gfsA*+pAN8.1	*ΔgfsAC*+*gfsA*	This study
MA886.1	MA880.1+*gfsC*+pAN8.1	*ΔgfsAC*+*gfsC*	This study

## Generation of *A. nige*r deletion mutants

The *A. niger gfsA, gfsB, and gfsC* genes were deleted by replacing their respective open-reading frames (ORFs) with the *A. oryzae pyrG* resistance cassette using the split marker approach as was described in detail by Arentshorst et al. 2015. Approximately 800 bp flanking regions of each of the ORFs were PCR amplified from genomic DNA of the N402 strain using primer pairs as listed in Additional file 1: Table 1. The *AopyrG* gene was amplified from pAO4-13 (de Ruiter-Jacobs et al. 1989), using primers AOpyrGP12f and AOpyrGP13r (Additional file 1: Table 1). Subsequently, 5′ and 3′ split marker fragments were obtained in two separate fusion PCR amplifications using the respective flank and the *AopyrG* PCR products as a template and primer pairs according to Additional file 1: Table 1. The split marker fragments were transformed to *A. niger* strain MA169.4 (Carvalho et al. 2010) and homologous integration was confirmed by Southern blot analysis (data not shown). Double mutants (*ΔgfsAB*, *ΔgfsAC,* and *ΔgfsBC)* were generated by transforming single mutants (*ΔgfsA* for *ΔgfsAB* and *ΔgfsC* for *ΔgfsAC* and *ΔgfsBC)* with split marker fragments containing hygromycin as selection marker (Arentshorst et al. 2015). To create a triple deletion mutant, a *pyrG*^*−*^ mutant of the *ΔgfsBC* strain was obtained from a 5-fluoroorotic acid plate. This strain (*ΔgfsBC, pyrG*^*−*^*)* was subsequently transformed with *ΔgfsA-AOpyrG* split marker fragments, resulting in a triple deletion mutant *(ΔgfsABC).* Proper deletion of the *gfs* genes in the respective deletion mutants was verified by Southern blot analysis (data not shown).

Complementation of the *Δgfs* mutants was performed by transforming the PCR amplified *gfs* genes, including ~ 800 bp promoter and ~ 800 bp termination regions, to the *gfs* deletion strains by cotransformation with the phleomycin resistance marker on pAN8.1 (Punt and Hondel 1992). To allow ectopic integration of the *gfs* genes, strains DL1.1 (*ΔgfsA)*, DL4.1 (*ΔgfsAC),* and DL5.1 (*ΔgfsAB)* were cured for their disruption of *ku70* by selection on 5′fluoro-acetamide to loop out the *amdS* marker used for disrupting *ku70* (Carvalho et al. 2010). For the amplification of the genes, the *gfs*-specific P1 and P4 primers were used (Additional file 1: Table 1). Phleomycin-resistant transformants were purified and analysed by diagnostic PCR to confirm the expected deletion and the presence of an ectopically integrated *gfs* gene. PCR-positive transformants were further analysed for their sensitivity towards CFW using the CFW spot assay.

## RT-qPCR experiments

Total RNA was extracted using TRIzol reagent (Invitrogen) from mycelium samples after growing the strains for 25 h in CM. RNA samples were further column purified using NucleoSpin RNA Clean-up kit (Macherey–Nagel) with rDNase treatment. The quantity and quality of the RNA samples were checked with a NanoDrop-1000 spectrophotometer (Thermo Fisher Scientific) and RNA gel electrophoresis, respectively. Primers for *agsA* and *actA* were designed using Primer-BLAST (Additional file 2: Table 2) (Ye et al. 2012). cDNA was synthesized using QuantiTect Reverse Transcription Kit (QIAGEN) according to the manufacturer’s instructions with 1 µg RNA per 20 µl total reaction volume and diluted afterwards 100 times. No reverse transcriptase samples, in which water was used instead of Reverse Transcriptase, were included to check for genomic DNA contamination. For each primer pair, efficiency of the reaction was calculated by generating a standard curve using cDNA obtained from 10 µg RNA per 200 µl total reaction volume and diluted to produce 10, 1, 0.1 and 0.01 ng RNA points. RNA obtained from *ΔugmA* strain grown for 25 h was used for standard curve generation. qPCR was carried out in a C1000 CFX96 machine (BIO-RAD) with 20 µl total reaction volume containing 2 µl cDNA, 10 µl 2 × GoTaq qPCR Master Mix (Promega), 6 µl water, 1 µl 5 µM forward primer, and 1 µl 5 µM reverse primer. In no template control samples, water was used instead of cDNA. 96-well white-shell white-well PCR plate (Hard-Shell PCR Plates, BIO-RAD) and optically clear adhesive seals (Microseal ‘B’ seal, BIO-RAD) were used. Each reaction was performed in three technical replicates. The protocol of qPCR was as follows: 2 min 50 °C, 10 min 95 °C, 50 cycles of 15 s 95 °C, 30 s 60 °C, and 30 s 72 °C. Melting curves were generated by increasing the temperature from 65 °C to 95 °C gradually. Specificity of reactions and contamination was checked for each primer pair. Data was analyzed using the accompanying software Bio-Rad CFX Manager 3.1 Expression values (ΔΔCq) were normalized against that of the reference gene *actA* and represented relative to the wild-type strain.

## Results and discussion

### Identification of three Galf-transferases in the* A. niger *genome

**Fig. 1:**
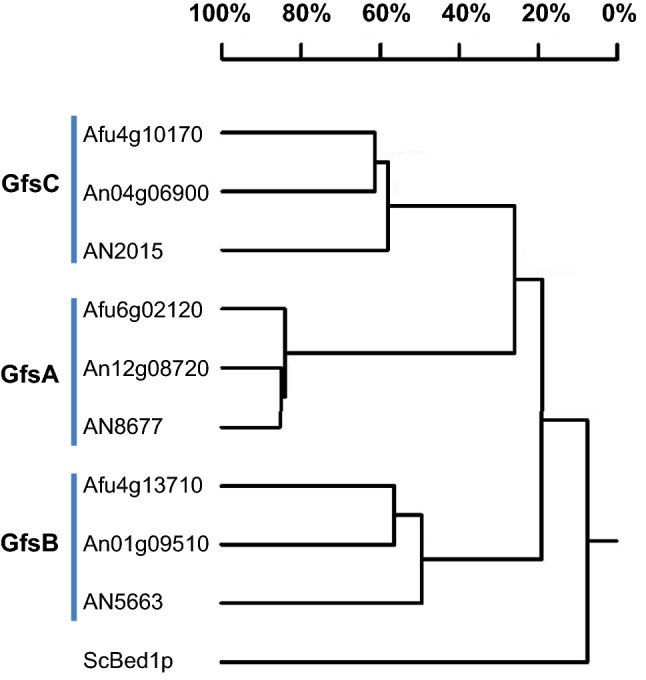
Phylogenetic tree of putative galactofuranosyltransferase from *A. niger*, *A. nidulans,* and *A. fumigatus.* Protein sequences were retrieved from AspGD (https://www.aspergillusgenome.org) and DNAman2.0 was used to make the homology tree. % of homology between the proteins is indicated. *Saccharomyces cerevisiae* galactotransferase Bed1p (Mnn10p) was used as an outgroup

Gal*f*-transferases in the genome of *A. niger* were identified by BlastP searches using the *A. nidulans* and *A. fumigatus* GfsA proteins as queries. We identified three putative homologs which were named GfsA, GfsB, and GfsC. *A. nidulans* and *A. fumigatus* also contain two additional candidates for Gal*f*-transferases, as noticed previously (Komachi et al. 2013; Oka and Goto 2016). Phylogenetic analysis showed that the three orthologs cluster in distinct clades (Fig. 1) indicating an early triplication of this gene family. The three *A. niger* candidates (GfsA: An12g08720, GfsB: An01g09510 and GfsC: An04g06900) are all predicted to be type II transmembrane proteins [Center for Biological Sequence analysis (https://www.cbs.dtu.dk/services/TMHMM/)] and are of about 500 amino acids in length (Table 2). Whereas the *ugmA/glfA* genes and the *ugtA/glfB* genes are clustered in the genome (Engel et al. 2009), the location of any of the three candidate Gal*f*-transferases in the genome was not clustered with other genes involved in Gal*f* biosynthesis.

**Table 2:** Characteristics of putative Galf-transferase *A. niger*

An number	Protein name	length (aa)	TM domain^a^	Probability TM prediction^a^
An12g08720	GfsA	532	20–37	0.99759
An01g09510	GfsB	568	13–35	0.97105
An04g06900	GfsC	461	7–24	0.91848

^a^Center for Biological Sequence analysis (https://www.cbs.dtu.dk/services/TMHMM/)

## Functional analysis of the putative Galf-transferases

**Fig. 2:**
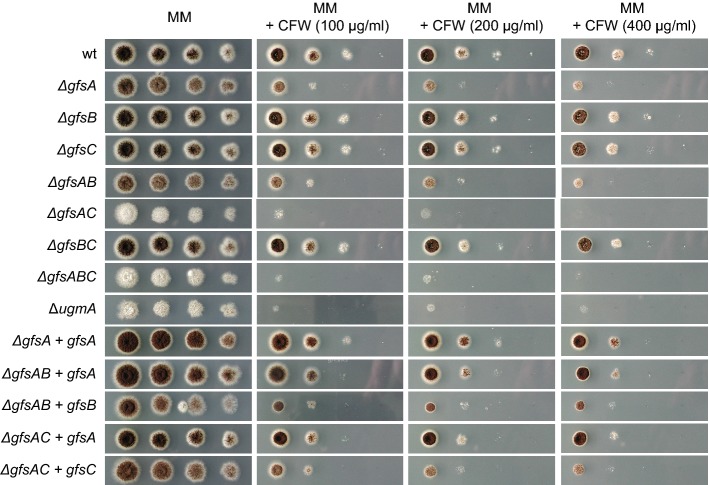
Phenotypic analysis of *gfsA* mutants. Tenfold dilutions of spores, starting with 1 × 10^4^ spores, were spotted on MM-agar or MM-agar + CFW (100 μg/ml) and incubated for 3 days at 30 °C. Figure is composed of several plates, incubated in parallel under identical conditions

To analyze the function of the different putative Gal*f*-transferases, *ΔgfsA*, *ΔgfsB,* and *ΔgfsC* single mutants, *ΔgfsAB*, *ΔgfsAC,* and *ΔgfsBC* double mutants, and a *ΔgfsABC* triple mutant were generated (Table 1), using the split marker method, with either the *A. oryzae pyrG* gene or the hygromycin resistance gene as a selection marker and MA169.4 (*ku70*^*−*^) as a host. The absence of galactofuranosylation, e.g., in the *ΔugmA* mutant, has been shown to result in a reduced growth phenotype, aberrant branching morphology, reduced conidiation, and increased sensitivity towards the cell wall assembly disturbing drug Calcofluor white (CFW) (Damveld et al. 2008; Park et al. 2016). Similar phenotypes were also observed in the *A. niger ΔugeA* mutant (Park et al. 2014) and the *ΔugtAB* double mutant (Park et al. 2015). When analyzing the growth phenotype of the different *Δgfs* mutants, we noticed, in the *ΔgfsA* single mutant, a reduced growth and an increased sensitivity towards CFW, although not as severe as in the *ΔugmA* mutant (Fig. 2). Deletion of *gfsC* alone did not result in an increased sensitivity to CFW; however, simultaneous deletion of *gfsA* and *gfsC* resulted in a severe phenotype, identical to the growth phenotypes of the *ΔugmA* strain, indicating that simultaneous deletion of *gfsA* and *gfsC* results in a complete galactofuranosylation defect. Deletion of *gfsB* does not seem to have an effect on the growth behaviour in the wild-type background as well as in combination with the deletion of *gfsA* and/or *gfsC*. To show that the deletions of *gfsA* and *gfsC* were responsible for the phenotypes of the single and double mutants, the mutants were complemented by transformation of the respective genes to the deletion mutants which restored the CFW sensitivity (Fig. 2).

**Fig. 3:**
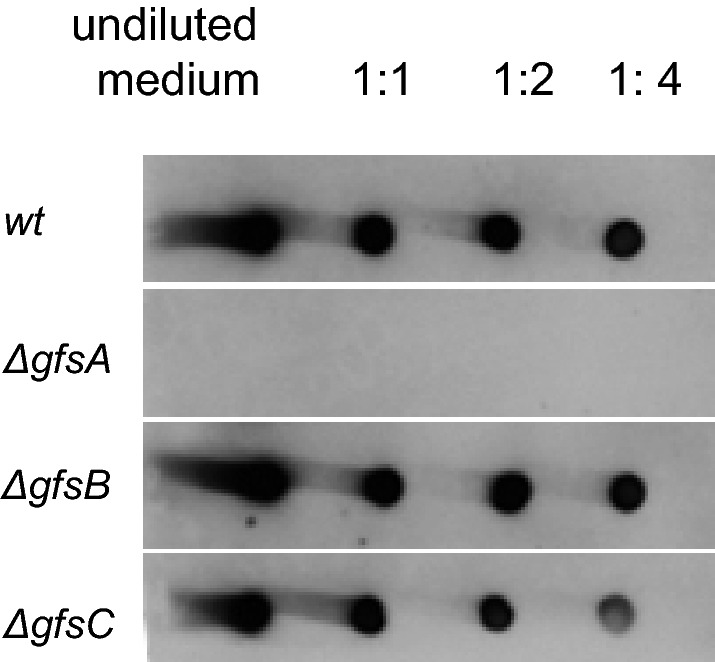
Dot-blot assay of Gal*f*-containing glycoconjugates in wild-type and *gfsA* mutants. *A. niger* wild-type strain and *gfs* mutants were grown in CM to early stationary phase (24 h) and cell-free medium was spotted on nitrocellulose filter paper. The blots were incubated with the anti-Gal*f* antibody (L10) to detect the presence of Gal*f*

To analyze the effect of the *gfsA*, *gfsB,* and *gfsC* deletion on the presence of Gal*f*-containing glycoconjugates in the growth medium, medium samples were spotted in nitrocellulose membrane and labelled with a Gal*f*-specific antibody (L10) as described (Park et al. 2014). As shown in Fig. 3, deletion of *gfsA* results in the absence of detectable amounts of Gal*f*. Based on the growth phenotype of the *ΔgfsA* mutant, however, which is not as severe as the *ugmA* mutant, it seems that some galactofuranosylation still occurs in the absence of *gfsA*. In the dot-blot experiment, it is likely that Gal*f*-residues on *N*- and *O*-glycans are detected. Therefore, the absence of detectable Gal*f* in the *ΔgfsA* mutant suggests that GfsA is required for the galactofuranosylation of *N-* and *O-*glycans.

## Activation of the cell wall integrity pathway in gfs-deficient mutants

**Fig. 4:**
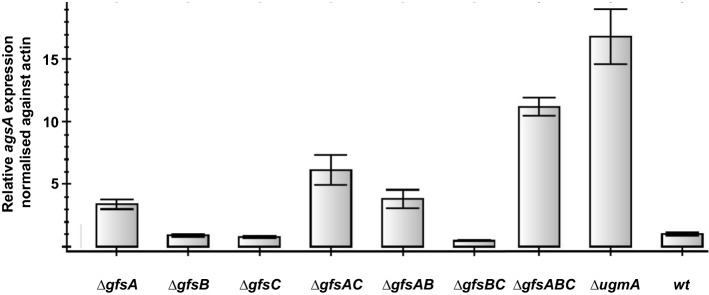
RT-qPCR analysis of *agsA* expression in single, double, and triple *Δgfs* strains, the *ΔugmA* mutant, and the wild-type (wt) strain. All strains were grown for 24 h at 30 °C, RNA was isolated and *agsA* expression was determined via RT-qPCR, using *actA* expression as reference. The *agsA* expression in all mutants is relative to the *agsA* expression in the wild-type strain, which was set to 1

The galactofuranose-deficient *ugeA* and *ugmA* mutants were identified in a screen for cell wall mutants with increased expression of the alpha-glucan synthase (*agsA*) (Damveld et al. 2008; Park et al. 2014). To identify additional mutants that are defective in Gal*f* biosynthesis, our collection of 240 mutants with induced expression of *agsA* was screened for lack of Gal*f* in the culture medium. However, screening of the collection failed to identify the *gfsA* mutant. Since the *gfsA* mutant is negative in the dot-blot assay (Fig. 3), we anticipated that a *gfsA* mutant could in principle be isolated in the mutant screen, if the *agsA* gene is strongly induced in the *gfsA* mutant. To analyze whether deletion of *gfsA* results in strong induction of the *agsA* gene, all single, double, and triple deletion strains as well as the *ΔugmA* strain were grown in liquid cultures for 25 h at 30 °C and RNA was isolated. The *agsA* expression in the mutants was determined by performing RT-qPCR experiments on these RNA samples, using *actA* expression as reference (Fig. 4). The RT-qPCR results show that the *agsA* expression in the *ΔugmA* strain is about fourfold higher than in the *ΔgfsA* strain, indicating that *agsA* induction in the *ΔgfsA* strain was probably not sufficient to be detected in the screen for cell wall mutants. Double deletion of both *gfsA* and *gfsC* as well as deletion of all three *gfs* genes causes a higher *agsA* induction, indicating again a redundant function of the *gfs* genes for the synthesis of Gal*f-*containing glycostructures in *A. niger* and activation of the cell wall stress response when multiple *gfs* genes are inactive.

## Conclusions

The biosynthesis of cell surface-located galactofuranose (Gal*f*)-containing glycostructures such as galactomannan, *N*-glycans, *O*-glycans, and glycolipids in filamentous fungi is important to secure the integrity of the cell wall. *A. niger* as well as *A. nidulans* and *A. fumigatus* contain three galactofuranosyltransferases encoding genes in their genomes. By constructing single, double, or triple *gfs* mutants and comparing the phenotype to the *ugmA* mutant, we show that GfsA together with GfsC are most important for galactofuranosylation in *A. niger.* The next step in our understanding of the function of the different galactofuranosyltransferases will be to elucidate whether individual genes are involved in the galactofuranosylation of the different glycostructures (galactomannan, *N*-glycans, *O*-glycans, and glycolipids) which contain Gal*f*.

## Electronic supplementary material

Below is the link to the electronic supplementary material.

Supplementary file1 (DOCX 14 kb)

Supplementary file2 (DOCX 12 kb)

## Abbreviations

Gal*f*: Galactofuranose
CFW: Calcofluor white
MM: Minimal medium
CM: Complete medium
