# Supplementary material for: Functional analysis of three putative galactofuranosyltransferases with redundant functions in galactofuranosylation in Aspergillus niger
Source: Arch Microbiol. 2019 Aug 1;202(1):197–203. doi: 10.1007/s00203-019-01709-w (PMC6949202; doi:10.1007/s00203-019-01709-w)
Supplement: Supplementary file 1 — Supplementary file1 (DOCX 14 kb) [file 203_2019_1709_MOESM1_ESM.docx]

**Additional file 1: Table 1: Primers used for cloning and sequencing**

| **Primer name** | **Sequence 5’-3’** | **Used for** |
| --- | --- | --- |
| An12g08720P1f | TGGTCCAGCATTCGGCAT | 5’ *gfsA* PCR |
| An12g08720P2r | **CAATTCCAGCAGCGGCTT**TTTGGCGGTATTATTATGAGTA | 5’ *gfsA* PCR |
| An12g08720P3f | **ACACGGCACAATTATCCATCG**AGTCCTTGGTCATGCAACTC | 3’ *gfsA* PCR |
| An12g08720P4r | AGGCCGTAGCCAGGTCAATC | 3’ *gfsA* PCR |
| An12g08720P5f | CAAAGCCTGCGATGTGGAC | Sequencing *gfsA* |
| An12g08720P6r | CTGCTTGGGAATGTCGTCG | Sequencing *gfsA* |
| An12g08720P7f | TGGACATGAAGGGTGAGGTTG | Sequencing *gfsA* |
| An01g09510P1f | AAGGAGAGCCGCACGGTTA | 5’ *gfsB* PCR |
| An01g09510P2r | **CAATTCCAGCAGCGGCTT**CATGGATCAGTCATGACGGT | 5’ *gfsB* PCR |
| An01g09510P3f | **ACACGGCACAATTATCCATCG**GATGACTGTTTTGATTAGATAG | 3’ *gfsB* PCR |
| An01g09510P4r | TCTCTCCAGACAAGCTCGGC | 3’ *gfsB* PCR |
| An01g09510P5f | CTATGTCGCTCCAATTCCCG | Sequencing *gfsB* |
| An01g09510P6r | CAAGCGCTCGTTATCTTCCTG | Sequencing *gfsB* |
| An01g09510P7f | ATTCCGCTTC TCGAGCAGC | Sequencing *gfsB* |
| An04g06900P1f | AGAGTGTCACCGTCCATCGC | 5’ *gfsC* PCR |
| An04g06900P2r | **CAATTCCAGCAGCGGCTT**TCTCGATTTTCCACTTCTTTCA | 5’ *gfsC* PCR |
| An04g06900P3f | **ACACGGCACAATTATCCATCG**GATTGGCCGCCTGTCTGC | 3’ *gfsC* PCR |
| An04g06900P4r | TCGCAGCTCTTACCAACTCCA | 3’ *gfsC* PCR |
| An04g06900P5f | GCTCTCCGCCGCTTAAATG | Sequencing *gfsC* |
| An04g06900P6r | TTCAGGACGCATATTCTTGGC | Sequencing *gfsC* |
| An04g06900P7f | GATATGATGGGCGATGTGTCC | Sequencing *gfsC* |
| AOpyrGP12f | **AAGCCGCTGCTGGAATTG** | *pyrG* marker PCR |
| AOpyrGP13r | **CGATGGATAATTGTGCCGTGT** | *pyrG* marker PCR |
| AOpyrGP14f | ATTGACCTACAGCGCACGC | *pyrG* split marker fusion PCR |
| AOpyrGP15r | CCGGTAGCCAAAGATCCCTT | *pyrG* split marker fusion PCR |
| hygP6for | **AAGCCGCTGCTGGAATTG**GGCTCTGAGGTGCAGTGGAT | hph marker PCR |
| hygP7rev | **CGATGGATAATTGTGCCGTGT**TGGGTGTTACGGAGCATTCA | hph marker PCR |
| hygP8for | AAAGTTCGACAGCGTCTCC | hph split marker fusion PCR |
| hygP9rev | GGCGTCGGTTTCCACTATC | hph split marker fusion PCR |

* overlapping sequences for fusion PCR are indicated in bold.
