# Supplementary material for: Functional analysis of three putative galactofuranosyltransferases with redundant functions in galactofuranosylation in Aspergillus niger
Source: Arch Microbiol. 2019 Aug 1;202(1):197–203. doi: 10.1007/s00203-019-01709-w (PMC6949202; doi:10.1007/s00203-019-01709-w)
Supplement: Supplementary file 2 — Supplementary file2 (DOCX 12 kb) [file 203_2019_1709_MOESM2_ESM.docx]

Additional file 2: Table 2: Primers used in RT-qPCR experiments.

| **Primer Name** | **Sequence (5' to 3')** | **Target** | **Amplicon Length** |  |
| --- | --- | --- | --- | --- |
| actA_C1f_qPCR | GTCTGGAGAGCGGTGGTATC | *actA* (An15g00560) | 110 bp | |
| actA_C1r_qPCR | GGTAGTACCACCAGACATGACA |  |  |  |
| agsAP34f_qPCR | GGTCATATGAGGAACGGATCAC | *agsA* (An04g09890) | 124 bp | |
| agsAP35r_qPCR | AGATCGACACCCTTCTGCTT |  |  |  |
